# A Gradient-Based Method for Robust Sensor Selection in Hypothesis Testing

**DOI:** 10.3390/s20030697

**Published:** 2020-01-27

**Authors:** Ting Ma, Bo Qian, Dunbiao Niu, Enbin Song, Qingjiang Shi

**Affiliations:** 1 College of Mathematics, Sichuan University, Chengdu 610064, China; majiawan27@163.com (T.M.); dunbiaoniu_sc@163.com (D.N.); 2 School of Electronic Science and Engineering, Nanjing University, Nanjing 210023, China; boqian@smail.nju.edu.cn; 3 School of Software Engineering, Tongji University, Shanghai 201804, China; shiqj@tongji.edu.cn

**Keywords:** wireless sensor network, robust sensor selection, hypothesis testing, Chernoff distance, Danskin’s theorem, orthogonal constraint-preserving gradient algorithm

## Abstract

This paper considers the binary Gaussian distribution robust hypothesis testing under a Bayesian optimal criterion in the wireless sensor network (WSN). The distribution covariance matrix under each hypothesis is known, while the distribution mean vector under each hypothesis drifts in an ellipsoidal uncertainty set. Because of the limited bandwidth and energy, we aim at seeking a subset of *p* out of *m* sensors such that the best detection performance is achieved. In this setup, the minimax robust sensor selection problem is proposed to deal with the uncertainties of distribution means. Following a popular method, minimizing the maximum overall error probability with respect to the selection matrix can be approximated by maximizing the minimum Chernoff distance between the distributions of the selected measurements under null hypothesis and alternative hypothesis to be detected. Then, we utilize Danskin’s theorem to compute the gradient of the objective function of the converted maximization problem, and apply the orthogonal constraint-preserving gradient algorithm (OCPGA) to solve the relaxed maximization problem without 0/1 constraints. It is shown that the OCPGA can obtain a stationary point of the relaxed problem. Meanwhile, we provide the computational complexity of the OCPGA, which is much lower than that of the existing greedy algorithm. Finally, numerical simulations illustrate that, after the same projection and refinement phases, the OCPGA-based method can obtain better solutions than the greedy algorithm-based method but with up to 48.72% shorter runtimes. Particularly, for small-scale problems, the OCPGA -based method is able to attain the globally optimal solution.

## 1. Introduction

Wireless sensor networks (WSNs) are extensively used to collect and transmit data in many applications, such as autonomous driving [1], disaster detection [2], target tracking [3], etc. In the WSN, it is usually unaffordable to collect and process all sensor data due to the limitations of power and communication resources [4,5]. Therefore, it is of great significance to choose an optimal subset of sensors such that the best performance is attained only based on data collected by the selected sensors, which is the so-called sensor selection problem.

In the past dozen years, sensor selection has been widely studied in various fields, e.g., estimation [6], target tracking [7], condition monitoring [8], to name a few. For parameter estimation in Kalman filtering dynamic system, [6] chose the optimal subset of sensors in each iteration via minimizing the error covariance matrix of the next iteration. The sensor selection problem for target tracking in large sensor networks was addressed in [7] based on generalized information gain. In [8], it provided an entropy-based sensor selection method for condition monitoring and prognostics of aircraft engine, which can describe the information contained in the sensor data sets.

Meanwhile, the sensor selection problem in hypothesis testings has also attracted a lot of attention [9,10,11]. For this type of hypothesis testings, only part of sensors in WSN are activated to transmit observation data, and then decisions are made based on the measurements of selected sensors to achieve the best detection performance. When the optimal sensor selection matrix is fixed, the corresponding hypothesis testing problem is reduced to a common one, which is easy to be dealt with. Hence, it is crucial to solve the involved sensor selection problem.

Work [9] studied the sensor selection for the binary Gaussian distribution hypothesis testing in the Neyman–Pearson framework, where the true distribution under each hypothesis is exactly known. It approximately converted the minimization of the false alarm error probability to the maximization of the Kullback-Leibler (KL) divergence between the distributions of the selected measurements under null hypothesis and alternative hypothesis to be detected. Additionally, [9] proposed a sensor selection framework of first relaxation and then projection for the first time, and provided the greedy algorithm to solve the relaxed problem by optimizing each column vector of the selection matrix.

In practical applications, the events to be detected (i.e., parameters of the hypothesis testing) are usually estimated from training data and affected by some uncertainty factors, such as poorly observation environment and system errors. Then, these parameters are not known precisely, but assumed to lie in some given uncertainty sets [10,11]. In these scenarios, the minimax robust sensor selection problem is formulated to cope with the parameter uncertainty. For the binary Gaussian distribution hypothesis testing under the Neyman–Pearson framework, following the framework in [9], work [10] investigated the involved sensor selection problem with distribution mean under each hypothesis falling in an ellipsoidal uncertainty set (the distribution covariance is known). Furthermore, [11] considered the sensor selection problems involved in the Gaussian distribution robust hypothesis testings with both Neyman–Pearson and Bayesian optimal criteria, where the distribution mean under each hypothesis drifts in an ellipsoidal uncertainty set. For the Bayesian framework, minimizing the maximum overall error probability is approximately converted to maximizing the minimum Chernoff distance. Then the corresponding greedy algorithm together with projection and refinement is also proposed to solve the robust sensor selection problem in the above hypothesis testing.

It has been shown in [11] that the robust sensor selection problem in the hypothesis testing is NP-hard under the Bayesian framework. Nevertheless, when the size of the sensor selection problem is small, its optimal solution can be obtained by the exhaustive method via traversing all possible choices. However, for a large-scale problem, the exhaustive method is not affordable due to its huge computation complexity. Although the aforementioned greedy algorithm-based method (i.e., greedy algorithm, projection and refinement) admits a lower computation complexity than the exhaustive method, it can not arrive at the globally optimal solution in many cases, and its computation complexity is still high for large-scale problems. Therefore, it is significant to seek a more efficient algorithm for solving the robust sensor selection problem in the hypothesis testing of WSN. To our surprise, even though other general sensor selection problems have been continuously investigated, for instance, sparse sensing [12] and sensor selection in sequential hypothesis testing [13], there is little progress for this type of sensor selection problems since [11] was published in 2011, which motivates our research.

In this paper, we consider the same binary Gaussian distribution robust hypothesis testings under a Bayesian framework as in [11], where the distribution mean under each hypothesis lies in an ellipsoidal uncertainty set. We attempt to select an optimal subset of sensors such that the maximum overall error probability is minimized. Following the similar idea in [11], minimizing the maximum overall error probability is approximated by maximizing the minimum Chernoff distance between two distributions under null hypothesis and alternative hypothesis to be detected. Then, our main contributions can be summarized as follows.

- First, we succeed in converting the maximinimization of the Chernoff distance to a maximization problem, and adopting the orthogonal constraint-preserving gradient algorithm (OCPGA) [14] to obtain a stationary point of the relaxed maximization problem without 0/1 constraints.
- Specifically, when implementing the OCPGA to the relaxed maximization problem, we utilize the Danskin’s theorem [15] to acquire its gradient. Furthermore, the efficient bisection is applied to get the means of distributions under null hypothesis and alternative hypothesis to be detected corresponding to the minimum Chernoff distance.
- The computational complexity of the OCPGA is shown to be lower than that of the greedy algorithm in [11] from the theoretical point of view, while numerical simulations show that the OCPGA-based method (i.e., OCPGA, projection and refinement) can obtain better solutions than the greedy algorithm-based method (i.e., R-C algorithm in [11]) with up to 48.72% shorter runtimes. Therefore, better solutions are available for our proposed OCPGA-based method in some scenarios.

The remainder of this paper is organized as follows. Section 2 states the problem formulation. The proposed OCPGA, as well as projection and refinement phases, is characterized in Section 3. In Section 4, the existence and computation of gradient are provided. Section 5 presents some numerical experiments to corroborate our theoretical results, while Section 6 concludes the paper.

*Notations*: Denote $R^{m}$ and $R^{m\times p}$ as the *m*-dimensional real vector space and m×p dimensional real matrix space, respectively. Let I and 0 be the identity matrix and the zero matrix whose dimensions will be clear from the context; bold-face lower-case letters are used for vectors, while bold-face upper-case letters are for matrices. N(μ,S) represents the Gaussian distribution with mean μ and covariance S. For matrix A, tr(A), |A|, ${∥A∥}_{F}$, $A^{H}$ and $A_{i,j}$ denote its trace, determinant, Frobenius norm, conjugate transpose and the (i,j)-th entry, respectively. For square matrices A and B, A⪰(≻)B represents that A–B is semi-positive (positive) definite. For a semi-positive definite matrix A, $A^{\frac{1}{2}}$ stands for its square-rooting matrix.

## 2. Problem Formulation

### 2.1. System Model

Define $x={\left(x_{1},x_{2},\cdots ,x_{m}\right)}^{T}\in R^{m}$ as the observation vector of all *m* sensors (each sensor corresponds to one-dimensional measurement). Consider the same binary Gaussian distribution robust hypothesis testing as in [11]:

(1) $$\begin{array}{c} H_{0}:\;x∼N\left(m_{0},S_{0}\right),\;\;\;\;\;\;H_{1}:\;x∼N\left(m_{1},S_{1}\right), \end{array}$$

where mean vector $m_{i}$ falls in a given ellipsoidal uncertainty set $E\left({\bar{m}}_{i},k_{i}S_{i}^{-1}\right)=\left\{x\in R^{m}|{\left(x-{\bar{m}}_{i}\right)}^{T}{S_{i}}^{-1}\left(x-{\bar{m}}_{i}\right)⩽\frac{1}{k_{i}}\right\}$, ${\bar{m}}_{i}$ denotes the mean estimated by training data, covariance $S_{i}$ is a known matrix, and $k_{i}\in \left(0,+\infty \right]$ is the robustness parameter, i=0,1. Obviously, when $k_{i}=+\infty$, the ellipsoidal uncertainty set is reduced to a single point, and thereby $m_{i}={\bar{m}}_{i}$, i.e., there is no uncertainty.

***Sensor Selection:*** In the WSN, sensors transmit their observations to a fusion node, which then performs the hypothesis testing based on its received measurements. Due to power constraints, suppose that only *p* out of *m* sensors are chosen (p<m) to transmit the observations to the fusion node. We aim at selecting *p* sensors to guarantee the best detection performance, that is, seeking a selection matrix $E\in R^{m\times p}$ with 0/1 elements such that the best detection performance based on measurements $y=E^{T}x\in R^{p}$ is achieved. It is easy to see that, E has exactly one unit entry per column (corresponds to a selected sensor) and at most one unit entry per row (each sensor is selected at most once). Therefore, E should be a column orthogonal matrix, i.e., $E^{T}E=I.$

***Hypothesis Testing Induced by E***: Owing to $y=E^{T}x$, the original hypothesis testing (1) about x in the high dimensional space $R^{m}$ is converted to one about y in a lower-dimensional space $R^{p}$:

(2) $$\begin{array}{c} H_{0}:\;y∼N\left(E^{T}m_{0},E^{T}S_{0}E\right),\;\;\;\;\;\;H_{1}:\;y∼N\left(E^{T}m_{1},E^{T}S_{1}E\right). \end{array}$$

Without loss of generality, we assume that

(3) $$N\left(E^{T}m_{0},E^{T}S_{0}E\right)\neq N\left(E^{T}m_{1},E^{T}S_{1}E\right).$$

Otherwise, the hypothesis testing (2) makes no sense. For hypothesis testing (2), when E and $m_{i}$ are determined, the fusion node executes the following likelihood ratio (LR) test:

$$l\left(y\right):=\frac{f_{1}\left(y;E\right)}{f_{0}\left(y;E\right)}\overset{H_{1}}{\underset{H_{0}}{≷}}\gamma ,$$

where $f_{i}\left(y;E\right)$ is the density of $N(E^{T}m_{i},E^{T}S_{i}E)$, i=0,1, and γ is the test threshold [16].

***Sensor Selection Optimality Criteria:*** Under a Bayesian framework, detection performance is quantified by the overall error probabilities $P_{e}$, where $P_{e}:=P\left(H_{0}\right)P_{F}+P\left(H_{1}\right)P_{M}$ with $P_{F}:=P\left(l\left(y\right)>\gamma |H_{0}\right)$ and $P_{M}:=P\left(l\left(y\right)<\gamma |H_{1}\right)$ being the false alarm and the miss detection probabilities respectively. Then, the robust sensor selection problem in the hypothesis testing under a Bayesian framework is to seek the selection matrix E such that the maximum overall error probability $P_{e}$ is minimized when making decisions with respect to hypothesis testing (2), that is, solving the following optimization problem:

(4) $$\begin{array}{cc} \; & \min_{E\in R^{m\times p}}\max_{\left(m_{0},m_{1}\right)\in E\left({\bar{m}}_{0},k_{0}{S_{0}}^{-1}\right)\times E\left({\bar{m}}_{1},k_{1}{S_{1}}^{-1}\right)}\;\;P_{e}\\ \; & \;\;s.t.\;\;\;\;\;\;E^{T}E=I,\;E_{i,j}\in \left\{0,1\right\}. \end{array}$$

### 2.2. Problem Transformation

Since computing $P_{e}$ in problem (4) is usually difficult due to the involved integrals, we follow the popular approach in [11] to approximately optimize $P_{e}$. Based on the Chernoff lemma [17], when the number of independent identically distributed (i.i.d.) measurements increases, the rate of exponential decay of $P_{e}$ is equal to the Chernoff distance between the two distributions $N(E^{T}m_{0},E^{T}S_{0}E)$ and $N(E^{T}m_{1},E^{T}S_{1}E)$. On the other hand, according to the definition of Chernoff distance between two probability densities $f_{0}$ and $f_{1}$:

$$D_{C}\left(f_{1},f_{0}\right):=\max_{s\in [0,1]}\log\int f_{1}^{s}\left(x\right)f_{0}^{1-s}\left(x\right)dx,$$

we derive that the Chernoff distance between distributions $N(E^{T}m_{0},E^{T}S_{0}E)$ and $N(E^{T}m_{1},E^{T}S_{1}E)$ is

(5) $$f_{C}\left(E,m_{0},m_{1}\right):=\max_{s\in [0,1]}f\left(E,s,m_{0},m_{1}\right)$$

with

(6) $$\begin{array}{cc} f(E,s,m_{0},m_{1}):= & \frac{1}{2}s\left(1-s\right){\left(m_{1}-m_{0}\right)}^{T}E{\left(E^{T}\left(sS_{0}+\left(1-s\right)S_{1}\right)E\right)}^{-1}E^{T}\\ \; & \times \left(m_{1}-m_{0}\right)-\frac{1}{2}\log\frac{|E^{T}S_{0}E{|}^{s}{\left|E^{T}S_{1}E\right|}^{1-s}}{|E^{T}\left(sS_{0}+\left(1-s\right)S_{1}\right)E|}. \end{array}$$

Therefore, as in [11], minimizing the maximum overall error probability $P_{e}$ can be approximately converted to maximizing the minimum Chernoff distance $f_{C}\left(E,m_{0},m_{1}\right)$. Accordingly, problem (4) is transformed into

(7) $$\begin{array}{cc} \; & \max_{E}\min_{\left(m_{0},m_{1}\right)\in E\left({\bar{m}}_{0},k_{0}{S_{0}}^{-1}\right)\times E\left({\bar{m}}_{1},k_{1}{S_{1}}^{-1}\right)}\;\;f_{C}\left(E,m_{0},m_{1}\right)\\ \; & \;\;s.t.\;\;\;\;\;\;\;\;\;\;E^{T}E=I,\;E_{i,j}\in \left\{0,1\right\}, \end{array}$$

or equivalently,

(8) $$\begin{array}{cc} \; & \max_{E}\;\min_{\left(m_{0},m_{1}\right)\in E\left({\bar{m}}_{0},k_{0}{S_{0}}^{-1}\right)\times E\left({\bar{m}}_{1},k_{1}{S_{1}}^{-1}\right)}\max_{s\in [0,1]}\;\;f\left(E,s,m_{0},m_{1}\right)\\ \; & \;\;s.t.\;\;\;\;\;\;\;\;\;E^{T}E=I,\;E_{i,j}\in \left\{0,1\right\}. \end{array}$$

Work [11] has proven that problem (7) is NP-hard, and proposed a suboptimal greedy method along with projection and refinement phases to deal with problem (7). Although the greedy algorithm-based method (i.e., R-C algorithm in [11]) admits a lower computation complexity than the exhaustive method, it can not arrive at the globally optimal solution in many cases, and still remains high computation complexity for large-scale problems. Therefore, we endeavor to propose a more efficient method to obtain a better solution of problem (7).

It is not difficult to see that, solving problem (7) can be sequentially divided into the inner minimization and outer maximization. For a given selection matrix E, defining

(9) $${\tilde{f}}_{C}\left(E\right):=\min_{\left(m_{0},m_{1}\right)\in E\left({\bar{m}}_{0},k_{0}{S_{0}}^{-1}\right)\times E\left({\bar{m}}_{1},k_{1}{S_{1}}^{-1}\right)}\;f_{C}\left(E,m_{0},m_{1}\right),$$

and denoting $\left({\hat{m}}_{0}\left(E\right),{\hat{m}}_{1}\left(E\right)\right)$ as the optimal solution of the following subproblem

(10) $$\min_{\left(m_{0},m_{1}\right)\in E\left({\bar{m}}_{0},k_{0}{S_{0}}^{-1}\right)\times E\left({\bar{m}}_{1},k_{1}{S_{1}}^{-1}\right)}\;f_{C}\left(E,m_{0},m_{1}\right),$$

then it holds ${\tilde{f}}_{C}\left(E\right)=f_{C}\left(E,{\hat{m}}_{0}\left(E\right),{\hat{m}}_{1}\left(E\right)\right)$. Correspondingly, problem (7) can be equivalently transformed into

(**PP**) Primal Problem (PP):maxE∈Rm×pf˜C(E)s.t.ETE=I,Ei,j∈{0,1}.

Remarkably, the optimal solutions of problems (7) and (**PP**) are the same. Hence, we will discuss how to solve problem (**PP**) in the following. Taking the orthogonal constraint $E^{T}E=I$ into account, we adopt the OCPGA-based method to deal with problem (**PP**).

## 3. OCPGA-Based Method

**Figure 1:**
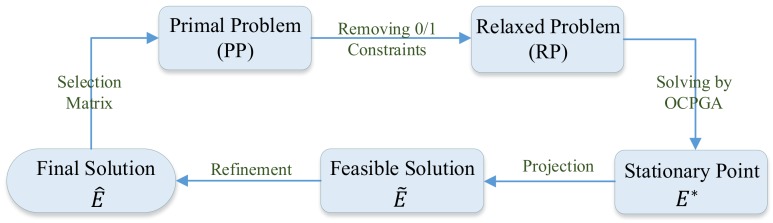
Working Flow of the OCPGA Based Method for Solving Problem (11).

Referring to the greedy algorithm-based method proposed in [11], solving problem (**PP**) can be also successively divided into three phases: relaxation, projection and refinement. Then we will utilize the OCPGA-based method to solve problem (**PP**). That is, in the relaxation phase the OCPGA is used to handle the relaxed problem without 0/1 constraints, while the projection and refinement phases are the same as in the greedy algorithm-based method. First we provide the working flow of the OCPGA-based method for solving problem (**PP**) in Figure 1, and the details are shown in the following Section 3.1 and Section 3.2.

### 3.1. Relaxation Phase

By relaxing the 0/1 constraints, problem (**PP**) is reduced to

(RP) Relaxed Problem (RP):maxE∈Rm×pf˜C(E)s.t.ETE=I.

Taking into consideration the orthogonal constraint in problem (**RP**), once the gradient $\nabla {\tilde{f}}_{C}\left(E\right)$ of the objective function ${\tilde{f}}_{C}\left(E\right)$ exists and is computable, we can implement the OCPGA in [14] to solve problem (**RP**), which is presented in Algorithm 1.
**Algorithm 1: OCPGA**
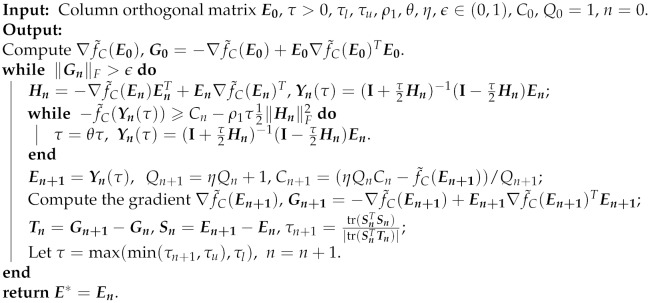


The update formula of $Y_{n}\left(\tau \right)$ in Algorithm 1 is the Cayley transformation [18], and thereby $Y_{n}\left(\tau \right)$ always satisfies the orthogonal constraint $Y_{n}{\left(\tau \right)}^{T}Y_{n}\left(\tau \right)=I$ for each iteration *n*. Meanwhile, the stepsize τ in Algorithm 1 is chosen by curvilinear search algorithms [19] combined with the Barzilai-Borwein (BB) [20] nonmonotonic line search [21]. It has been shown by Lemma 2.2 and Remark 2.3 in [22] that the sequence generated by the OCPGA is globally convergent to a stationary point. For clarity and integrity, we provide the convergence result of Algorithm 1 in the following theorem without proof.

**Theorem** **1** ([22])**.** When ${\tilde{f}}_{C}\left(E\right)$ in problem (***RP***) is differentiable and the gradient $\nabla {\tilde{f}}_{C}\left(E\right)$ is derived, then $E^{*}$ obtained by the OCPGA in Algorithm 1 is a stationary point of problem (***RP***).

Moreover, the computation complexity of the OCPGA in Algorithm 1 is $O(mp^{2})$ [14]. Combined with the fact that *p* is generally much smaller than *m*, the computation complexity of the OCPGA is much lower than that $O(m^{3}p)$ of the greedy algorithm in [11]. Hence, our proposed OCPGA is more efficient than the greedy algorithm, particularly for a quite large *m*.

### 3.2. Projection and Refinement Phases

Generally, the solution $E^{*}$ obtained by the OCPGA in Algorithm 1 is not a selection matrix, because the elements of $E^{*}$ are not guaranteed to be 0/1. Thus we need to further execute the projection and refinement phases as in [11].

First, we seek matrix $\tilde{E}$ closest to the range space of $E^{*}$ by solving the following problem

(11) $$\begin{array}{cc} \min_{E}\;\; & ∥EE^{T}-E^{*}{\left(E^{*}\right)}^{T}{∥}_{F}\\ s.t.\;\; & E_{i,j}\in \left\{0,1\right\},\;E^{T}E=I. \end{array}$$

It has been shown in [11] that problem (11) admits a closed-form solution. Specifically, let $\left(j_{1},\cdots ,j_{p}\right)$ be the indexes of the *p* largest entries on the diagonal of $E^{*}{\left(E^{*}\right)}^{T}$, and then $\tilde{E}=\left(i_{j_{1}},\cdots ,i_{j_{p}}\right)$ is the optimal solution of problem (11), where $i_{j}$ stands for the *j*-th column of the identity matrix $I_{m}$.

Subsequently, after projecting to the set of 0/1 selection matrices, we further implement a refinement around $\tilde{E}$. Setting $E=\tilde{E}$, the first column of E is viewed as the optimization variable, while all other columns are fixed. Then we sweep through all canonical vectors (i.e., columns of the identity matrix $I_{m}$) different from the remaining p−1 columns of E, and choose the one corresponding to the maximum ${\tilde{f}}_{C}$ as the first column. In the next step, the procedure is repeated for the second column, and so on, up to the *p*-th step. Finally, one refinement is finished and the matrix $\hat{E}=E$ is regarded as a solution of problem (**PP**). Obviously, the more we refine, the better solution we can achieve. When p=1, the solution achieved by one refinement is indeed the globally optimal selection matrix of problem (**PP**). If time permits, we can execute the refinement phase for several times until the objective function value ${\tilde{f}}_{C}$ keeps unchanged.

Notice that, the computation cost for projection is very small (as there exists an analytical solution), while in refinement we need to calculate the objective function value ${\tilde{f}}_{C}$ for (m−p)p times. Since the OCPGA is more efficient than the greedy algorithm, and the remaining projection and refinement phases are the same, the OCPGA-based method naturally possesses higher efficiency than the greedy algorithm-based method.

**Remark** **1.** *For the OCPGA in Algorithm 1, its initial point is randomly chosen from column orthogonal matrices. Experiments illustrate that different initial points may result in different outcomes. Therefore, in order to improve the performance, we implement the OCPGA with different initial points several times, and after projection and refinement, choose the best solution as the output.*

**Remark** **2.** *According to the above discussion, the key to implement the OCPGA-based method is computing the gradient $\nabla {\tilde{f}}_{C}\left(E\right)$ in problem (***RP***). In the next section, we will show how to obtain the gradient $\nabla {\tilde{f}}_{C}\left(E\right)$ and discuss when it exists.*

## 4. Existence and Computation of the Gradient in Problem (RP)

As can be easily seen from Algorithm 1, it is essential to compute the gradient $\nabla {\tilde{f}}_{C}\left(E\right)$ in problem (**RP**). Invoking the definition of ${\tilde{f}}_{C}\left(E\right)$ in Equation (9), we have

$${\tilde{f}}_{C}\left(E\right)=\min_{\left(m_{0},m_{1}\right)\in E\left({\bar{m}}_{0},k_{0}{S_{0}}^{-1}\right)\times E\left({\bar{m}}_{1},k_{1}{S_{1}}^{-1}\right)}\;f_{C}\left(E,m_{0},m_{1}\right),$$

with $f_{C}\left(E,m_{0},m_{1}\right)=\max_{s\in [0,1]}f\left(E,s,m_{0},m_{1}\right)$. To proceed, we first prove the strict concavity of $f(E,s,m_{0},m_{1})$ with respect to *s*, which forms a key ingredient of our later arguments.

**Proposition** **1.** *Given E and $\left(m_{0},m_{1}\right)$, $f(E,s,m_{0},m_{1})$ defined by Equation* (6) *is a strictly concave function of s.*

**Proof.**  It is easily seen that $f(E,s,m_{0},m_{1})$ is continuously differentiable with respect to *s* for given E and $\left(m_{0},m_{1}\right)$. Hence we turn to show the positiveness of the second derivative $\nabla_{s}^{2}f\left(E,s,m_{0},m_{1}\right)$.

For notation brevity, define $\Lambda :=\left(m_{1}-m_{0}\right){\left(m_{1}-m_{0}\right)}^{T}$ and $B\left(s\right):=E{\left(E^{T}\left(sS_{0}+\left(1-s\right)S_{1}\right)E\right)}^{-1}E^{T}$. It is clear that both Λ and B(s) are semi-positive definite. By taking derivative of $f(E,s,m_{0},m_{1})$ with respect to *s*, we have

(12) $$\begin{array}{cc} \nabla_{s}f\left(E,s,m_{0},m_{1}\right)= & \left(\frac{1}{2}-s\right)\mathrm{tr}\left[B\left(s\right)\Lambda \right]+\frac{1}{2}\mathrm{tr}\left[B\left(s\right)\left(S_{0}-S_{1}\right)\right]\\ & -\frac{1}{2}s\left(1-s\right)\mathrm{tr}\left[B\left(s\right)\left(S_{0}-S_{1}\right)B\left(s\right)\Lambda \right]-\frac{1}{2}\log\frac{|E^{T}S_{0}E|}{|E^{T}S_{1}E|}. \end{array}$$

Then, taking derivative of $\nabla_{s}f\left(E,s,m_{0},m_{1}\right)$ with respect to *s* once again, we obtain

(13) $$\begin{array}{cc} \nabla_{s}^{2}f\left(E,s,m_{0},m_{1}\right)= & -\mathrm{tr}\left[B\left(s\right)\Lambda \right]+\left(2s-1\right)\mathrm{tr}\left[B\left(s\right)\left(S_{0}-S_{1}\right)B\left(s\right)\Lambda \right]\\ \; & \;+s\left(1-s\right)\mathrm{tr}\left\{{\left[B\left(s\right)\left(S_{0}-S_{1}\right)\right]}^{2}B\left(s\right)\Lambda \right\}-\frac{1}{2}\mathrm{tr}\left\{{\left[B\left(s\right)\left(S_{0}-S_{1}\right)\right]}^{2}\right\}\\ \overset{\left(a\right)}{=} & -\mathrm{tr}\left[B\left(s\right)\Lambda \right]+\left(s-1\right)\mathrm{tr}\left[B\left(s\right)\left(S_{0}-S_{1}\right)B\left(s\right)\Lambda \right]\\ & \;\;+s\;\mathrm{tr}\left[B\left(s\right)S_{0}B\left(s\right)\left(S_{0}-S_{1}\right)B\left(s\right)\Lambda \right]-\frac{1}{2}\mathrm{tr}\left\{{\left[B\left(s\right)\left(S_{0}-S_{1}\right)\right]}^{2}\right\}\\ \overset{\left(b\right)}{=} & -\frac{1}{1-s}\mathrm{tr}\left[B\left(s\right)S_{0}B\left(s\right)\Lambda \right]+\frac{s}{1-s}\mathrm{tr}\left[B\left(s\right)S_{0}B\left(s\right)S_{0}B\left(s\right)\Lambda \right]-\frac{1}{2}\mathrm{tr}\left\{{\left[B\left(s\right)\left(S_{0}-S_{1}\right)\right]}^{2}\right\}\\ \overset{\left(c\right)}{=} & -\frac{1}{1-s}\mathrm{tr}\left\{\Lambda^{\frac{1}{2}}B\left(s\right)S_{0}E\Gamma \left(s\right)E^{T}S_{0}B\left(s\right)\Lambda^{\frac{1}{2}}\right\}-\frac{1}{2}\mathrm{tr}\left\{{\left[B\left(s\right)\left(S_{0}-S_{1}\right)\right]}^{2}\right\}, \end{array}$$

where $\Gamma \left(s\right)={\left(E^{T}S_{0}E\right)}^{-1}-{\left(E^{T}S_{0}E+\frac{1-s}{s}E^{T}S_{1}E\right)}^{-1}$, equalities (a) and (b) both result from $B\left(s\right)=B\left(s\right)[sS_{0}+\left(1-s\right)S_{1}]B\left(s\right)$ and thus $\left(1-s\right)B\left(s\right)\left(S_{0}-S_{1}\right)B\left(s\right)=B\left(s\right)S_{0}B\left(s\right)-B\left(s\right)$, and equality (c) owing to the definition of B(s) and the relationship tr(AB)=tr(BA) for matrices A and B.

If $E^{T}m_{0}\neq E^{T}m_{1}$, then we have $E^{T}\Lambda^{\frac{1}{2}}\neq 0$. Because of $E^{T}S_{0}E≻0$, it holds

$$\begin{array}{c} E^{T}S_{0}B\left(s\right)\Lambda^{\frac{1}{2}}=E^{T}S_{0}E{\left[A\left(s\right)\right]}^{-1}E^{T}\Lambda^{\frac{1}{2}}\neq 0 \end{array}$$

with $A\left(s\right)=E^{T}\left(sS_{0}+\left(1-s\right)S_{1}\right)E≻0$. Combined with Γ(s)≻0, B(s)⪰0, and $\mathrm{tr}\left\{{\left[B\left(s\right)\left(S_{0}-S_{1}\right)\right]}^{2}\right\}=\mathrm{tr}\left\{B\left(s\right)\left[\left(S_{0}-S_{1}\right)B\left(s\right)\left(S_{0}-S_{1}\right)\right]\right\}⩾0$, we conclude $\nabla_{s}^{2}f\left(E,s,m_{0},m_{1}\right)<0$ from Equation (13).

On the other hand, when $E^{T}m_{0}=E^{T}m_{1}$, then it follows from Equation (3) that $E^{T}S_{0}E\neq E^{T}S_{1}E$. If ${\left[B\left(s\right)\left(S_{0}-S_{1}\right)\right]}^{2}=0$, Then we have $B\left(s\right)\left(S_{0}-S_{1}\right)B\left(s\right)=0$, that is

$$\begin{array}{c} E{\left[A\left(s\right)\right]}^{-1}\left(E^{T}S_{0}E-E^{T}S_{1}E\right){\left[A\left(s\right)\right]}^{-1}E^{T}=0. \end{array}$$

Then it immediately follows $E^{T}S_{0}E-E^{T}S_{1}E=0$, which leads to a contradiction. Therefore, when $E^{T}m_{0}=E^{T}m_{1}$, we deduce $\mathrm{tr}\left\{{\left[B\left(s\right)\left(S_{0}-S_{1}\right)\right]}^{2}\right\}>0$, which means $\nabla_{s}^{2}f\left(E,s,m_{0},m_{1}\right)<0$ by Equation (13).

As a consequence, $f(E,s,m_{0},m_{1})$ is a strictly concave function of *s*. □

Moreover, the following Proposition 2 shows that $f_{C}\left(E,m_{0},m_{1}\right)$ is continuously differentiable with respect to E for given $\left(m_{0},m_{1}\right)$, which is also necessary for follow-up analysis.

**Proposition** **2.** *Given $\left(m_{0},m_{1}\right)$, $f_{C}\left(E,m_{0},m_{1}\right)$ defined by* (5) *is a continuously differentiable function of E.*

**Proof.**  For given E and $\left(m_{0},m_{1}\right)$, denote $s^{*}:=\arg\max_{s\in [0,1]}f\left(E,s,m_{0},m_{1}\right)$ with $f(E,s,m_{0},m_{1})$ given by Equation (6). Note that $f\left(E,0,m_{0},m_{1}\right)=f\left(E,1,m_{0},m_{1}\right)=0$ and $f_{C}\left(E,m_{0},m_{1}\right)=\max_{s\in [0,1]}f\left(E,s,m_{0},m_{1}\right)>0$ under condition (3). Hence, $s^{*}\in \left(0,1\right)$, which, combined with the strict concavity of $f(E,s,m_{0},m_{1})$ in Proposition 1, implies that $s^{*}$ is unique with satisfying $\nabla_{s}f\left(E,s,m_{0},m_{1}\right)=0.$

Recalling the expression of $\nabla_{s}f\left(E,s,m_{0},m_{1}\right)$ in (12), since all the involved terms B(s), tr(·), |·|, log(·) and $E^{T}S_{0}E\neq 0$ are continuously differentiable functions of *E*, then $\nabla_{s}f\left(E,s,m_{0},m_{1}\right)$ as a composite function of the above functions is also continuously differentiable with respect to *E*. Similarly, $\nabla_{s}^{2}f\left(E,s,m_{0},m_{1}\right)$ in (13) is a composition of functions $\frac{1}{1-s}$, B(s) and tr(·), which are all continuous with respect to *s*. Hence, $\nabla_{s}^{2}f\left(E,s,m_{0},m_{1}\right)$ is continuous with respect to *s*, that is, $\nabla_{s}f\left(E,s,m_{0},m_{1}\right)$ is continuously differentiable with respect to *s*. Due to $\nabla_{s}^{2}f\left(E,s,m_{0},m_{1}\right)\neq 0$, it follows from the implicit function theorem [23] that $s^{*}$ is an implicit function of E. Hence, we rewrite $s^{*}$ as

(14) $$s^{*}\left(E\right):=\arg\max_{s\in [0,1]}f\left(E,s,m_{0},m_{1}\right).$$

Furthermore, based on the implicit function theorem [23], $s^{*}\left(E\right)$ is a continuously differentiable function of E, which means that $\nabla s^{*}\left(E\right)$ is continuous with respect to E.

Because of $f_{C}\left(E,m_{0},m_{1}\right)=\max_{s\in [0,1]}f\left(E,s,m_{0},m_{1}\right)$, we have $f_{C}\left(E,m_{0},m_{1}\right)=f\left(E,s^{*}\left(E\right),m_{0},m_{1}\right)$ immediately. In addition, $f(E,s,m_{0},m_{1})$ is continuously differentiable with *s* and E. Combined with the fact that $s^{*}\left(E\right)$ is a continuously differentiable function of E, thereby $f_{C}\left(E,m_{0},m_{1}\right)$ is differentiable with respect to E.

Moreover, by leveraging on the chain rule [24] to $f_{C}\left(E,m_{0},m_{1}\right)=f\left(E,s^{*}\left(E\right),m_{0},m_{1}\right)$, it holds

$$\begin{array}{cc} \; & \nabla_{E}f_{C}\left(E,m_{0},m_{1}\right)=\left[\nabla_{E}f\left(E,s,m_{0},m_{1}\right)+\nabla_{s}f\left(E,s,m_{0},m_{1}\right)\nabla s^{*}\left(E\right)\right]{|}_{s=s^{*}\left(E\right)}, \end{array}$$

where

(15) $$\begin{array}{cc} \nabla_{E}f\left(E,s,m_{0},m_{1}\right)\; & =s\left(1-s\right)\left[I-W\left(s\right)EA^{-1}\left(s\right)E^{T}\right]\Lambda EA^{-1}\left(s\right)\\ \; & \;\;\;\;-sS_{0}E{\left(E^{T}S_{0}E\right)}^{-1}-\left(1-s\right)S_{1}E{\left(E^{T}S_{1}E\right)}^{-1}+W\left(s\right)EA^{-1}\left(s\right) \end{array}$$

with $W\left(s\right)=sS_{0}+\left(1-s\right)S_{1}$, $A\left(s\right)=E^{T}W\left(s\right)E$, and $\Lambda =\left(m_{1}-m_{0}\right){\left(m_{1}-m_{0}\right)}^{T}$. Since $\nabla s^{*}\left(E\right)$, $\nabla_{s}f\left(E,s,m_{0},m_{1}\right)$ and $\nabla_{E}f\left(E,s,m_{0},m_{1}\right)$ are all continuous with respect to E, then $\nabla_{E}f_{C}\left(E,m_{0},m_{1}\right)$ is also continuous. In conclusion, $f_{C}\left(E,m_{0},m_{1}\right)$ is a continuously differentiable function of E. □

### 4.1. Compute the Gradient in Problem (***RP***) by Danskin’s Theorem

In the sequel, we will exploit Danskin’s theorem in Appendix A to compute the gradient $\nabla {\tilde{f}}_{C}\left(E\right)$, where ${\tilde{f}}_{C}\left(E\right)$ is defined by Equation (9).

On basis of Proposition 2, the following results hold true for function $f_{C}\left(E,m_{0},m_{1}\right)$ and set $E\left({\bar{m}}_{0},k_{0}{S_{0}}^{-1}\right)\times E\left({\bar{m}}_{1},k_{1}{S_{1}}^{-1}\right)$:

1. $E\left({\bar{m}}_{0},k_{0}{S_{0}}^{-1}\right)\times E\left({\bar{m}}_{1},k_{1}{S_{1}}^{-1}\right)$ is a compact set because it is a finite-dimensional bounded closed set. Meanwhile, $\forall \;(m_{0},m_{1})$, mapping $\left(t,m_{0},m_{1}\right)\to f_{C}\left(E+th,m_{0},m_{1}\right)$ is continuous at point $\left(0,m_{0},m_{1}\right)$ due to the continuity of $f_{C}\left(E,m_{0},m_{1}\right)$;
2. For arbitrary given $\left(m_{0},m_{1}\right)$ and sufficiently small t>0, since $f_{C}\left(E,m_{0},m_{1}\right)$ is differentiable, there exists a bounded directional derivative
  $$\begin{array}{c} D_{1}f_{C}\left(E+th,m_{0},m_{1};h\right)=\lim_{\tau \to 0^{+}}\frac{1}{\tau }\times \left[f_{C}\left(E+\left(t+\tau \right)h,m_{0},m_{1}\right)-f_{C}\left(E+th,m_{0},m_{1}\right)\right]; \end{array}$$
3. Mapping $\left(t,m_{0},m_{1}\right)\to D_{1}f_{C}\left(E+th,m_{0},m_{1}\right)$ is continuous at point $\left(0,m_{0},m_{1}\right)$, which is from the continuity of $\nabla_{E}f_{C}\left(E,m_{0},m_{1}\right)$.

With identifications E∼u, $(m_{0},m_{1})∼v$, $R^{m\times p}∼U$, $E\left({\bar{m}}_{0},k_{0}{S_{0}}^{-1}\right)\times E\left({\bar{m}}_{1},k_{1}{S_{1}}^{-1}\right)∼V$, $-f_{C}\left(E,m_{0},m_{1}\right)∼J\left(u,v\right)$, and $-{\tilde{f}}_{C}\left(E\right)∼\bar{J}\left(u\right)$, all conditions of Danskin’s theorem in Appendix A are satisfied. Subsequently, for a given selection matrix E, if the optimal solution $\left({\hat{m}}_{0}\left(E\right),{\hat{m}}_{1}\left(E\right)\right)$ of problem (10) is unique, then the gradient of ${\tilde{f}}_{C}\left(E\right)$ exists. Furthermore, on basis of the Danskin’s theorem, we have

(16) $$\nabla {\tilde{f}}_{C}\left(E\right)=\nabla_{E}f_{C}\left(E,m_{0},m_{1}\right){|}_{\left(m_{0},m_{1}\right)=\left({\hat{m}}_{0}\left(E\right),{\hat{m}}_{1}\left(E\right)\right)}.$$

Recall $f_{C}\left(E,m_{0},m_{1}\right)=\max_{s\in [0,1]}f\left(E,s,m_{0},m_{1}\right)$ from Equation (5). For given $\left(m_{0},m_{1}\right)$, we can again utilize the Danskin’s theorem in Appendix A to obtain $\nabla_{E}f_{C}\left(E,m_{0},m_{1}\right)$. Obviously, $f(E,s,m_{0},m_{1})$ is continuously differentiable with respect to E. Therefore, it is easy to verify that conditions 1)−3) of Danskin’s theorem in Appendix Appendix A are all satisfied with identifications E∼u, s∼v, $R^{m\times p}∼U$, [0,1]∼V, $f\left(E,s,m_{0},m_{1}\right)∼J\left(u,v\right)$, and $f_{C}\left(E,m_{0},m_{1}\right)∼\bar{J}\left(u\right)$. Combined with the uniqueness of $s^{*}\left(E\right)$ defined by Equation (14), we can deduce

(17) $$\nabla_{E}f_{C}\left(E,m_{0},m_{1}\right)=\nabla_{E}f\left(E,s,m_{0},m_{1}\right){|}_{s=s^{*}\left(E\right)}.$$

On the other hand, due to the equivalence of Problems (7) and (8), for a given matrix E, a solution $\left({\hat{m}}_{0}\left(E\right),{\hat{m}}_{1}\left(E\right)\right)$ of Problem (10) corresponds to a solution $\left(\hat{s}\left(E\right),{\hat{m}}_{0}\left(E\right),{\hat{m}}_{1}\left(E\right)\right)$ of problem

(**IP**) Iner Problem (IP):min(m0,m1)∈E(m¯0,k0S0−1)×E(m¯1,k1S1−1)maxs∈[0,1]f(E,s,m0,m1),

satisfying $f_{C}\left(E,{\hat{m}}_{0}\left(E\right),{\hat{m}}_{1}\left(E\right)\right)=f\left(E,\hat{s}\left(E\right),{\hat{m}}_{0}\left(E\right),{\hat{m}}_{1}\left(E\right)\right)$ and

(18) $$\hat{s}\left(E\right)=\arg\max_{s\in [0,1]}f\left(E,s,{\hat{m}}_{0}\left(E\right),{\hat{m}}_{1}\left(E\right)\right).$$

Combined with Equations (16) and (17), when the solution $\left(\hat{s}\left(E\right),{\hat{m}}_{0}\left(E\right),{\hat{m}}_{1}\left(E\right)\right)$ is unique, we have

(19) $$\begin{array}{cc} \; & \nabla {\tilde{f}}_{C}\left(E\right)=\nabla_{E}f\left(s,E,m_{0},m_{1}\right){|}_{\left(s,m_{0},m_{1}\right)=\left(\hat{s}\left(E\right),{\hat{m}}_{0}\left(E\right),{\hat{m}}_{1}\left(E\right)\right)}, \end{array}$$

where $\nabla_{E}f\left(s,E,m_{0},m_{1}\right)$ is given by Equation (15) and $\left(\hat{s}\left(E\right),{\hat{m}}_{0}\left(E\right),{\hat{m}}_{1}\left(E\right)\right)$ is the optimal solution of problem (**IP**).

### 4.2. Compute the Optimal Solution of Problem (***IP***)

Owing to the Sion’s minimax theorem [25] and Lemma 5 in [11], for given E, we can exchange the orders of the minimization with respect to $\left(m_{0},m_{1}\right)$ and the maximization with respect to *s* in problem (**IP**). Correspondingly, problem (**IP**) can be equivalently converted into

(20) $$\begin{array}{cc} \; & \max_{s\in [0,1]}\min_{\left(m_{0},m_{1}\right)\in E\left({\bar{m}}_{0},k_{0}{S_{0}}^{-1}\right)\times E\left({\bar{m}}_{1},k_{1}{S_{1}}^{-1}\right)}\;\;f\left(E,s,m_{0},m_{1}\right). \end{array}$$

Therefore, we will solve Problem (20) to attain the solution $\left(\hat{s}\left(E\right),{\hat{m}}_{0}\left(E\right),{\hat{m}}_{1}\left(E\right)\right)$ of Problem (**IP**).

First, for given matrix E and parameter *s*, denote

$$\left(m_{0}^{*}\left(s\right),m_{1}^{*}\left(s\right)\right):=\arg\min_{\left(m_{0},m_{1}\right)\in E\left({\bar{m}}_{0},k_{0}{S_{0}}^{-1}\right)\times E\left({\bar{m}}_{1},k_{1}{S_{1}}^{-1}\right)}\;\;f\left(E,s,m_{0},m_{1}\right).$$

Combining with the expression of $f(E,s,m_{0},m_{1})$ in Equation (6) and removing items irrelevant to $\left(m_{0},m_{1}\right)$, then we can get $\left(m_{0}^{*}\left(s\right),m_{1}^{*}\left(s\right)\right)$ by solving the following subproblem

(21) $$\begin{array}{c} \min_{\left(m_{0},m_{1}\right)\in E\left({\bar{m}}_{0},k_{0}{S_{0}}^{-1}\right)\times E\left({\bar{m}}_{1},k_{1}{S_{1}}^{-1}\right)}\;\;{\left(m_{1}-m_{0}\right)}^{T}B\left(m_{1}-m_{0}\right) \end{array}$$

with $B:=E{\left(E^{T}\left(sS_{0}+\left(1-s\right)S_{1}\right)E\right)}^{-1}E^{T}⪰0$. Clearly, Problem (21) is convex, which can be directly solved by the CVX toolbox [26] with computation complexity $O(p^{3})$ [27]. Thus, once *s* is fixed, the corresponding $\left(m_{0}^{*}\left(s\right),m_{1}^{*}\left(s\right)\right)$ follows. Particularly, $\left({\hat{m}}_{0}\left(E\right),{\hat{m}}_{1}\left(E\right)\right)=\left(m_{0}^{*}\left(\hat{s}\left(E\right)\right),m_{1}^{*}\left(\hat{s}\left(E\right)\right)\right)$.

Next, we will show how to determine the optimal $\hat{s}\left(E\right)$ given by Equation (18). Based on Proposition 1, $f(E,s,m_{0},m_{1})$ is concave with respect to *s*. Combined with the fact that $f(E,s,m_{0}^{*}\left(s\right),m_{1}^{*}\left(s\right))$ is the minimum of a family of functions $f(E,s,m_{0},m_{1})$ over the uncertainty set $E\left({\bar{m}}_{0},k_{0}{S_{0}}^{-1}\right)\times E\left({\bar{m}}_{1},k_{1}{S_{1}}^{-1}\right)$, we conclude that $f(E,s,m_{0}^{*}\left(s\right),m_{1}^{*}\left(s\right))$ is also a concave function of *s* [28]. Therefore, $\nabla_{s}f\left(E,s,m_{0}^{*}\left(s\right),m_{1}^{*}\left(s\right)\right)$ is monotonically decreasing with respect to *s*. Subsequently, we apply the efficient bisection method to search $\hat{s}\left(E\right)$ such that $\nabla_{s}f\left(E,\hat{s}\left(E\right),m_{0}^{*}\left(\hat{s}\left(E\right)\right),m_{1}^{*}\left(\hat{s}\left(E\right)\right)\right)=0.$

For given E, we can once again use the Danskin’s theorem in Appendix A to derive $\nabla_{s}f\left(E,s,m_{0}^{*}\left(s\right),m_{1}^{*}\left(s\right)\right)$. Since $f(E,s,m_{0},m_{1})$ is continuously differentiable with respect to *s*, all conditions of Danskin’s theorem in Appendix A are met. Meanwhile, even if the optimal solution $\left(m_{0}^{*}\left(s\right),m_{1}^{*}\left(s\right)\right)$ of Problem (21) is not unique, $\nabla_{s}f\left(E,s,m_{0}^{*}\left(s\right),m_{1}^{*}\left(s\right)\right)$ in Equation (12) is always the same no matter which $\left(m_{0}^{*}\left(s\right),m_{1}^{*}\left(s\right)\right)$ is substituted. Therefore, $f(E,s,m_{0}^{*}\left(s\right),m_{1}^{*}\left(s\right))$ is differentiable with respect to *s*, and we have

(22) $$\begin{array}{c} \nabla_{s}f\left(E,s,m_{0}^{*}\left(s\right),m_{1}^{*}\left(s\right)\right)=\nabla_{s}f\left(E,s,m_{0},m_{1}\right){|}_{\left(m_{0},m_{1}\right)=\left(m_{0}^{*}\left(s\right),m_{1}^{*}\left(s\right)\right)}, \end{array}$$

where $\nabla_{s}f\left(E,s,m_{0},m_{1}\right)$ is given by Equation (12), and $\left(m_{0}^{*}\left(s\right),m_{1}^{*}\left(s\right)\right)$ is an arbitrary optimal solution of Problem (21).

After we get the optimal $\hat{s}\left(E\right)$, the corresponding $\left(m_{0}^{*}\left(\hat{s}\right),m_{1}^{*}\left(\hat{s}\right)\right)$, i.e., $\left({\hat{m}}_{0}\left(E\right),{\hat{m}}_{1}\left(E\right)\right)$, is acquired by solving Problem (21). That is, the optimal solution $\left(\hat{s}\left(E\right),{\hat{m}}_{0}\left(E\right),{\hat{m}}_{1}\left(E\right)\right)$ of Problem (**IP**) is obtained. We summarize the process of solving $\left(\hat{s}\left(E\right),{\hat{m}}_{0}\left(E\right),{\hat{m}}_{1}\left(E\right)\right)$ as the following **Inner Procedure 1**.
**Inner Procedure 1: Computing the Optimal Solution of Problem (IP)**
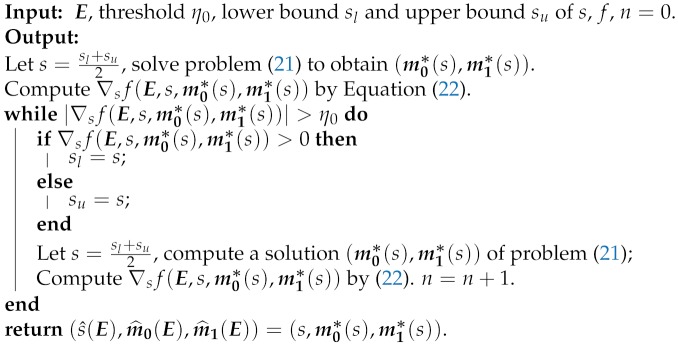

**Remark** **3.** If the true distribution under each hypothesis is exactly known, i.e., there is no uncertainty in the mean vector, then we omit the process of computing solution $\left(m_{0}^{*}\left(\hat{s}\right),m_{1}^{*}\left(\hat{s}\right)\right)$ of problem (21), and directly regard the true mean vector as $\left(m_{0}^{*}\left(\hat{s}\right),m_{1}^{*}\left(\hat{s}\right)\right)$. Consequently, the robust sensor selection problem is reduced to one without uncertainty.

Based on the previous discussion, if the optimal solution $\left(\hat{s}\left(E\right),{\hat{m}}_{0}\left(E\right),{\hat{m}}_{1}\left(E\right)\right)$ of Problem (**IP**) is unique, then the gradient $\nabla {\tilde{f}}_{C}\left(E\right)$ exists and can be computed by Equation (19). Next we will discuss the existence of the gradient $\nabla {\tilde{f}}_{C}\left(E\right)$ detailedly.

### 4.3. Existence of the Gradient in Problem (***RP***)

It has been shown that the uniqueness of the optimal solution $\left(\hat{s}\left(E\right),{\hat{m}}_{0}\left(E\right),{\hat{m}}_{1}\left(E\right)\right)$ leads to the existence of the gradient $\nabla {\tilde{f}}_{C}\left(E\right)$. Therefore, in the following, we turn to show when $\left(\hat{s}\left(E\right),{\hat{m}}_{0}\left(E\right),{\hat{m}}_{1}\left(E\right)\right)$ is unique. First the following lemma demonstrates the uniqueness of $\hat{s}\left(E\right)$.

**Lemma** **1.** *For given E, let $\left(\hat{s}\left(E\right),{\hat{m}}_{0}\left(E\right),{\hat{m}}_{1}\left(E\right)\right)$ be the optimal solution of Problem (***IP***). Then $\hat{s}\left(E\right)$ is unique.*

**Proof.**  We will prove by contradiction. Assume that $\left(\tilde{s}\left(E\right),{\tilde{m}}_{0}\left(E\right),{\tilde{m}}_{1}\left(E\right)\right)$ is also the optimal solution of problem (**IP**) with $\tilde{s}\left(E\right)\neq \hat{s}\left(E\right)$.

It is worth noting that, both $\left(\hat{s}\left(E\right),{\hat{m}}_{0}\left(E\right),{\hat{m}}_{1}\left(E\right)\right)$ and $\left(\tilde{s}\left(E\right),{\tilde{m}}_{0}\left(E\right),{\tilde{m}}_{1}\left(E\right)\right)$ are the saddle points of problem (**IP**). According to Proposition 1.4 in [29], the set of saddle points admits the Cartesian product form. Hence, $\left(\tilde{s}\left(E\right),{\hat{m}}_{0}\left(E\right),{\hat{m}}_{1}\left(E\right)\right)$ is also a saddle point. That is, for given $\left({\hat{m}}_{0}\left(E\right),{\hat{m}}_{1}\left(E\right)\right)$, both $\hat{s}\left(E\right)$ and $\tilde{s}\left(E\right)$ are the optimal solutions of problem $\max_{s\in [0,1]}f\left(E,s,{\hat{m}}_{0}\left(E\right),{\hat{m}}_{1}\left(E\right)\right)$, which contradicts with the strict concavity of $f(E,s,m_{0},m_{1})$ with respect to *s*. Consequently, $\hat{s}\left(E\right)$ is unique. □

With the uniqueness of $\hat{s}\left(E\right)$, we additionally make the following assumption to guarantee the existence of $\nabla {\tilde{f}}_{C}\left(E\right)$.

**Assumption** **1.** For given matrix E, the saddle point $\left(\hat{s}\left(E\right),m_{0}^{*}\left(\hat{s}\left(E\right)\right),m_{1}^{*}\left(\hat{s}\left(E\right)\right)\right)$ of Problem (***IP***) is unique.

**Remark** **4.** Because Lemma 1 has proven that $\hat{s}\left(E\right)$ is unique, if the solution $\left(m_{0}^{*}\left(\hat{s}\left(E\right)\right),m_{1}^{*}\left(\hat{s}\left(E\right)\right)\right)$ of Problem (21) is unique, then Assumption A1 is satisfied. Under some conditions (e.g., the distance of the two uncertainty sets $E({\bar{m}}_{0},k_{0}{S_{0}}^{-1})$ and $E({\bar{m}}_{1},k_{1}{S_{1}}^{-1})$ is large), $\left(m_{0}^{*}\left(\hat{s}\left(E\right)\right),m_{1}^{*}\left(\hat{s}\left(E\right)\right)\right)$ of Problem (21) *is naturally unique. Therefore, Assumption 1 is not very restrictive.*

Under Assumption 1, $\left(\hat{s}\left(E\right),{\hat{m}}_{0}\left(E\right),{\hat{m}}_{1}\left(E\right)\right)$ is unique and achievable by **Inner Procedure 1**. Hence, $\nabla {\tilde{f}}_{C}\left(E\right)$ exists and can be obtained by Equation (19). Subsequently, Algorithm 1 can be executed. Furthermore, on basis of Theorem 1 in Section 3.1, we conclude that $E^{*}$ obtained by the OCPGA in Algorithm 1 is a stationary point of problem (**RP**), which lays a foundation to attain a better solution of the original problem (**PP**).

**Remark** **5.** *If Assumption 1 is not satisfied, we could use the Clark generalized gradient [30] to replace the gradient in Algorithm 1. Based on the Danskin’s theorem in Appendix A, the Clark generalized gradient can also be attained by Equation* (19), *where an arbitrary solution of Problem* (21) *is used. Then Algorithm 1 is still applicable and preserves the orthogonal constraint in each iteration. Although the resulting solution is not guaranteed to be a stationary point of Problem (***RP***), however, after projection and refinement phases, numerical simulations illustrate that the performance of the final result is also acceptable.*

## 5. Numerical Simulations

In this section, numerical examples are carried out to show that the OCPGA-based method can obtain better solutions than the greedy algorithm-based method in [11]. To this end, (1) for fixed-size sensor selection problems, i.e., the total number and selected number of sensors are fixed, with randomly generated 50 (or 20) cases with different uncertainty sets, we exhibit the proportions that the OCPGA-based method performs better than, the same as, and worse than the greedy algorithm-based method; (2) for small-scale sensor selection problems, we compare the OCPGA-based method with the greedy algorithm based method and the exhaustive method; (3) for larger-scale sensor selection problems, the OCPGA-based method is compared with the greedy algorithm based method; (4) for specific small-scale and larger-scale sensor selection problems, the corresponding receiver operating characteristic (ROC) curves are depicted. Notably, in cases of (1)–(3), the performance of the method is measured by the resulting Chernoff distance (i.e., ${\tilde{f}}_{C}\left(E\right)$ in Problem (**PP**)), that is, the method with larger Chernoff distance admits better performance. All the procedures are coded in MATLAB R2014b on an ASUS notebook with the Intel(R) Core(TM) i3-2310M CPU of 2.10GHz and memory of 6GB.

Assume that we need to choose *p* out of *m* sensors. In all simulations, for given (m,p), the ellipsoidal uncertainty sets $E({\bar{m}}_{i},k_{i}{S_{i}}^{-1})$ in Problem (**IP**), i=0,1, which contain the true distribution under each hypothesis, are generated as follows. Elements of the estimated mean vectors ${\bar{m}}_{0}$ and ${\bar{m}}_{1}$ are randomly generated from (0,1) and (0,2), respectively. The covariance matrix $S_{i}$ is generated by $S_{i}={P_{i}\Sigma_{i}P_{i}}^{T}$, where $P_{i}$ is an orthogonal basis of m×m-dimensional matrices whose elements are generally drawn from (0,1), and $\Sigma_{i}$ is a diagonal matrix with diagonal entries randomly generated in (0,2), i=0,1. The robustness parameters $k_{0}=k_{1}=1$.

When (m,p) and the ellipsoidal uncertainty sets $E({\bar{m}}_{i},k_{i}{S_{i}}^{-1})$ are given, we adopt **Inner Procedure 1** to compute $\left(\hat{s}\left(E\right),{\hat{m}}_{0}\left(E\right),{\hat{m}}_{1}\left(E\right)\right)$, use Equation (19) to obtain the gradient $\nabla {\tilde{f}}_{C}\left(E\right)$, and then apply the OCPGA in Algorithm 1 to get the stationary point $E^{*}$ of Problem (RP). After the projection and refinement phases described in Section 3.2, the final solution $\hat{E}$ of Problem (**PP**) is achieved. Meanwhile, the greedy algorithm-based method is a deterministic approach, that is, for given (m,p) and ellipsoidal uncertainty set, its outputs are all the same no matter how many times it is recalled. On the contrary, since the initial point of the OCPGA in Algorithm 1 is randomly chosen, the outputs of the OCPGA-based method may vary with initial points of the OCPGA. If time permits, the OCPGA-based method can be recalled for several times to achieve better performance. Moreover, the OCPGA and the greedy algorithm based methods both execute one refinement only.

*Fixed-Size Examples:* For 8 fixed pairs of small (m,p), we give the proportions that the OCPGA-based method performs better than, the same as, and worse than the greedy algorithm-based method. For each (m,p), by implementing the two methods with randomly generated 50 different ellipsoidal uncertainty sets (only 20 different ellipsoidal uncertainty sets for (50,5), (80,5), and (100,5) due to their long runtimes), the corresponding results are listed in Table 1. Here, with each ellipsoidal uncertainty set, the OCPGA-based method is recalled for two times and the better result is selected as the output. As shown in Table 1, for each (m,p), the OCPGA-based method performs as well as the greedy algorithm-based method in most cases, while the “better” proportion is more than twice as many as the “worse” proportion. Actually, simulations show that, for “worse” cases, if we recall the OCPGA-based method for more times, then it can perform as well as even better than the greedy algorithm-based method.

*Small-Scale Examples:* we consider small-scale sensor selection problems, whose globally optimal solutions can be attained by the exhaustive method. Hence, we compare the optimal Chernoff distance obtained by the OCPGA-based method with those of the greedy algorithm-based method and the exhaustive method. Suppose that p=3,4,5 out of m=10,12,15 sensors are chosen. The corresponding outputs of the three methods are given in Table 2, and the corresponding runtimes of the three methods are listed in Table 3. It can be easily seen from Table 2 that, the Chernoff distances achieved by the OCPGA-based method are larger than those of the existing greedy algorithm-based method. For the case of (15,3), the Chernoff distance achieved by the OCPGA-based method is even 100% larger than that of the greedy algorithm-based method. In particular, our proposed OCPGA-based method can attain the same performance as the exhaustive method. Meanwhile, it is shown in Table 3 that, the OCPGA-based method is more efficient than the greedy algorithm-based method, and both of them possess much shorter runtimes than the exhaustive method, which is coincident with the theoretical computation complexity analyses. Via simple computations, we can see from Table 3 that the runtime of the OCPGA-based method can be up to 48.72% shorter than that of greedy algorithm-based method (for the case of (10,4)). Therefore, our proposed OCPGA-based method admits better performance in terms of not only the objective value but also the runtime.

*Larger-Scale Examples:* Now we consider larger-scale problems, where m=50,80,100 and p=5,10,15. Since *m* and *p* are large, which leads to the failure of the exhaustive method, we only compare the obtained Chernoff distances of our OCPGA-based method with those of the greedy algorithm-based method. The corresponding results are exhibited in Table 4, while the corresponding runtimes of the two methods are displayed in Table 5. As we can see from Table 4, the OCPGA-based method can attain a better solution than the greedy algorithm-based method. In the case of (50,5), the Chernoff distance obtained by the OCPGA-based method can be 13.14% larger than that of the greedy algorithm-based method. Moreover, Table 5 illustrates that, the OCPGA-based method admits higher efficiency than the greedy algorithm -based method, and the runtime of the OCPGA-based method can reduce up to 42.19% in the case of (50,5). Compared with small-scale cases in Table 3, we can see from Table 5 that the improvement in runtime is more obvious, which is due to the larger gap between *m* and *p* for larger-scale cases. Hence, for larger-scale cases, the OCPGA-based method also can achieve better solutions than the greedy algorithm-based method with shorter runtimes.

**Figure 2:**
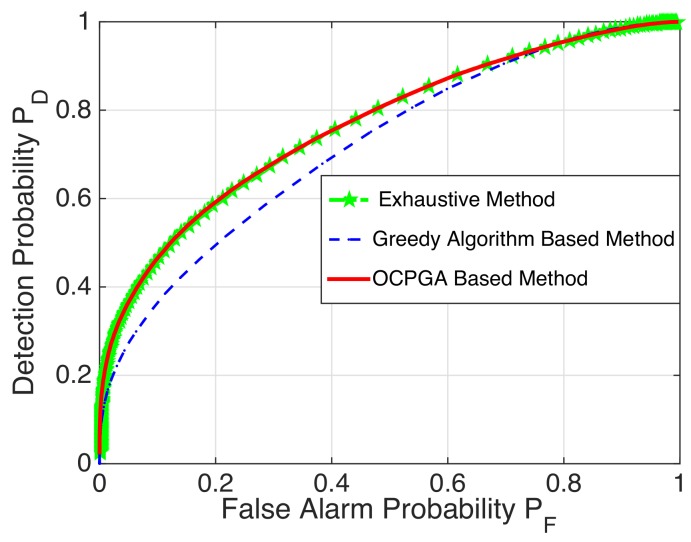
ROC curve for the small-scale case.

**Figure 3:**
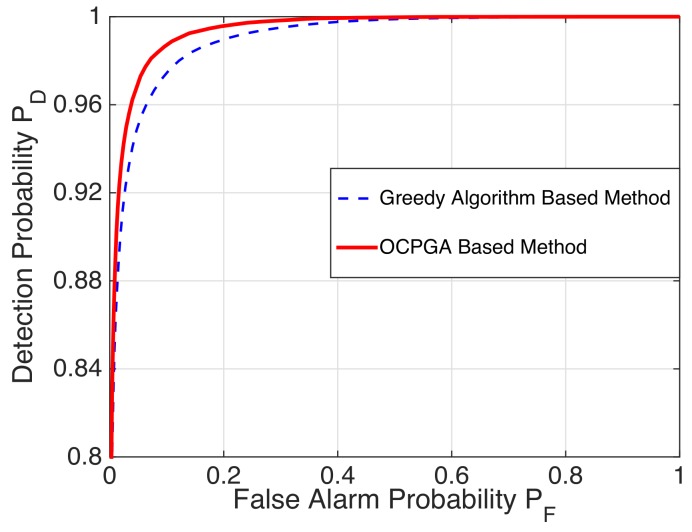
ROC curve for the larger-scale case.

*ROC Curves for Specific Examples:* By Monte Carlo simulations with 200,000 instantiations of the LR tests and calculating $P_{D}/P_{F}$ with $P_{D}:=1-P_{M}$ being the detection probability, we depict the ROC curves to show the validity of the OCPGA-based method. It is well known that the higher the ROC curve, the better the detection performance. For the case of (m,p)=(10,3) in Table 2, we display the corresponding ROC curves of the exhaustive method, the greedy algorithm-based method and the OCPGA-based method. It can be seen from Figure 2 that the OCPGA-based method is superior to the greedy algorithm-based method, while it can attain the same performance as that of the exhaustive method. Similarly, for the case of (m,p)=(50,5) in Table 4, Figure 3 illustrates that our proposed approach performs better than the existing greedy algorithm-based method.

In summary, compared with the greedy algorithm-based method, the OCPGA-based method not only admits a lower theoretical computation complexity, but also can obtain better solutions with shorter runtimes in numerical simulations.

## 6. Conclusions

We address the minimax robust sensor selection in the binary Gaussian distribution hypothesis testing of WSN with the distribution mean vector under each hypothesis drifting in an ellipsoidal uncertainty set. Under a Bayesian optimal criterion, minimizing the maximum overall error probability with respect to the selection matrix is approximately converted to maximizing the minimum Chernoff distance between the distributions under a null hypothesis and alternative hypothesis to be detected. Then, the gradient of the objective function of the converted maximization problem is computed by Danskin’s theorem. Furthermore, we apply the OCPGA to solve the relaxed maximization problem without 0/1 constraints, which can get a stationary point of the relaxed problem with lower computational complexity than the existing greedy algorithm. Numerical simulations demonstrate that the OCPGA-based method can attain better solutions than the greedy algorithm-based method with up to 48.72% shorter runtimes, and the OCPGA-based method is able to attain the globally optimal solution obtained by the exhaustive method for small-scale problems. In future work, we can consider cases where the distribution mean falls in other types of uncertainty sets such as the band model. In addition, the cases with not precisely known distribution covariance can also be a future research direction.

## Figures and Tables

**Table 1:** Performance Comparison Proportions.

(m,p)	Better	Same	Worse
(10,3)	20%	74%	6%
(10,4)	24%	72%	4%
(10,5)	20%	76%	4%
(12,3)	18%	74%	8%
(15,3)	22%	74%	4%
(50,5)	20%	70%	10%
(80,5)	15%	80%	5%
(100,5)	10%	85%	5%

**Table 2:** Optimal Chernoff Distances of Three Methods.

*m*	p=3			p=4			p=5		
	Exh	Gre	OCP	Exh	Gre	OCP	Exh	Gre	OCP
10	0.15	0.11	0.15	0.34	0.28	0.34	0.29	0.26	0.29
12	0.51	0.35	0.51	0.25	0.21	0.25	0.62	0.34	0.62
15	0.30	0.15	0.30	0.77	0.76	0.77	0.20	0.19	0.20

Exh: Exhaustive Method; Gre: Greedy Algorithm Based Method; OCP: OCPGA Based Method.

**Table 3:** Runtimes of Three Methods (s).

*m*	p=3			p=4			p=5		
	Exh	Gre	OCP	Exh	Gre	OCP	Exh	Gre	OCP
10	2.59$\times 10^{3}$	52.12	51.07	1.10$\times 10^{4}$	204.77	105.01	6.49 $\times 10^{4}$	87.07	80.39
12	4.17$\times 10^{3}$	128.75	109.35	2.66$\times 10^{4}$	82.41	75.94	2.04$\times 10^{5}$	106.53	98.31
15	4.33$\times 10^{4}$	92.68	89.28	1.50$\times 10^{5}$	193.45	188.95	1.20$\times 10^{6}$	153.09	150.41

Exh: Exhaustive Method; Gre: Greedy Algorithm Based Method; OCP: OCPGA Based Method.

**Table 4:** Optimal Chernoff Distances of Two Methods.

*m*	p=5		p=10		p=15	
	Gre	OCP	Gre	OCP	Gre	OCP
50	1.37	1.55	3.59	3.68	3.82	3.85
80	1.32	1.34	3.36	3.41	5.86	5.92
100	1.69	1.71	4.46	4.56	5.74	6.06

Gre: Greedy Algorithm Based Method; OCP: OCPGA Based Method.

**Table 5:** Runtimes of Two Methods (s).

*m*	p=5		p=10		p=15	
	Gre	OCP	Gre	OCP	Gre	OCP
50	1499.76	866.96	2083.39	1956.96	3794.17	3635.68
80	1839.27	1718.73	5435.69	5251.96	5350.99	5240.56
100	2290.26	1752.71	5589.49	5273.85	11966.38	11433.33

Gre: Greedy Algorithm Based Method; OCP: OCPGA Based Method.

## Abbreviations

The following abbreviations are used in this manuscript:

WSN	Wireless sensor network
KL	Kullback-Leibler
LR	Likelihood ratio
OCPGA	Orthogonal constraint-preserving gradient algorithm
PP	Primal Problem
RP	Relaxed Problem
IP	Inner Problem
ROC	Receiver operating characteristic

## Appendix A. Danskin’s Theorem (Theorem D1 and Its Corollary [15])

Let *U* and *V* be subsets of a Banach space U and a topological space V respectively. *J* is a mapping from U×V into R. Assume $\bar{J}\left(u\right)=\underset{v\in V}{\sup}J\left(u,v\right)$, and $\hat{V}\left(u\right)=v\in V|J\left(u,v\right)=\bar{J}\left(u\right)$. Moreover, suppose the following conditions hold true

(1) *V* is compact, ∀v∈V, the application (t,v)→J(u+th,v) is upper semi-continuous at (0,v);
(2) ∀v∈V and ∀t in a right neighborhood of 0, there exists a bounded directional derivative
  $$\begin{array}{cc} \; & D_{1}J\left(u+th,v;h\right)=\lim_{\tau \to 0^{+}}\frac{1}{\tau }\left[J\left(u+\left(t+\tau \right)h,v\right)-J\left(u+th,v\right)\right]; \end{array}$$
(3) the map $\left(t,v\right)\to D_{1}J\left(u+th,v\right)$ is upper semi-continuous at (0,v).

Then the function $\bar{J}$ has a directional derivative at *u* in the direction *h*, given by the formula

$$D\bar{J}\left(u;h\right)=\max_{v\in \hat{V}\left(u\right)}D_{1}J\left(u,v;h\right)\}.$$

Moreover, If u→J(u,v) has a Gateaux derivative $J_{u}^{\prime }$, and if the max is unique: $\hat{V}\left(u\right)=\left\{\hat{v}\right\}$, then $\bar{J}$ has a Gateaux derivative ${\bar{J}}^{\prime }\left(u\right)$ given by the simple formula

$${\bar{J}}^{\prime }\left(u\right)=J_{u}^{\prime }\left(u,\hat{v}\right).$$
